# EEG-based graph network analysis in relation to regional tau in asymptomatic Alzheimer’s disease

**DOI:** 10.1093/braincomms/fcaf138

**Published:** 2025-04-15

**Authors:** Laure Spruyt, Tjaša Mlinarič, Nathalie Dusart, Mariska Reinartz, Gabriela Meade, Marc M Van Hulle, Koen Van Laere, Patrick Dupont, Rik Vandenberghe

**Affiliations:** Laboratory for Cognitive Neurology, Department of Neurosciences, KU Leuven, Leuven 3000, Belgium; Alzheimer Research Centre KU Leuven, Leuven Brain Institute (LBI), KU Leuven, Leuven 3000, Belgium; Alzheimer Research Centre KU Leuven, Leuven Brain Institute (LBI), KU Leuven, Leuven 3000, Belgium; Laboratory for Neuro- and Psychophysiology, Department of Neurosciences, Leuven Brain institute, KU Leuven, Leuven 3000, Belgium; Laboratory for Cognitive Neurology, Department of Neurosciences, KU Leuven, Leuven 3000, Belgium; Alzheimer Research Centre KU Leuven, Leuven Brain Institute (LBI), KU Leuven, Leuven 3000, Belgium; Laboratory for Cognitive Neurology, Department of Neurosciences, KU Leuven, Leuven 3000, Belgium; Alzheimer Research Centre KU Leuven, Leuven Brain Institute (LBI), KU Leuven, Leuven 3000, Belgium; Division of Speech Pathology, Department of Neurology, Mayo Clinic, Rochester, MN 55905, USA; Alzheimer Research Centre KU Leuven, Leuven Brain Institute (LBI), KU Leuven, Leuven 3000, Belgium; Laboratory for Neuro- and Psychophysiology, Department of Neurosciences, Leuven Brain institute, KU Leuven, Leuven 3000, Belgium; Alzheimer Research Centre KU Leuven, Leuven Brain Institute (LBI), KU Leuven, Leuven 3000, Belgium; Nuclear Medicine and Molecular Imaging, Department of Imaging and Pathology, KU Leuven, Leuven 3000, Belgium; Division of Nuclear Medicine, UZ Leuven, Leuven 3000, Belgium; Laboratory for Cognitive Neurology, Department of Neurosciences, KU Leuven, Leuven 3000, Belgium; Alzheimer Research Centre KU Leuven, Leuven Brain Institute (LBI), KU Leuven, Leuven 3000, Belgium; Laboratory for Cognitive Neurology, Department of Neurosciences, KU Leuven, Leuven 3000, Belgium; Alzheimer Research Centre KU Leuven, Leuven Brain Institute (LBI), KU Leuven, Leuven 3000, Belgium; Department of Neurology, UZ Leuven, Leuven 3000, Belgium

**Keywords:** ^18^F-MK6240 PET, ^18^F-NAV4694 PET, ^11^C-PiB PET, functional connectivity, high-density EEG

## Abstract

Tau aggregation in early affected regions in the asymptomatic stage of Alzheimer’s disease marks a transitional phase between stable asymptomatic amyloid positivity and the clinically manifest stage. How this early region tau aggregation covertly affects brain function during this asymptomatic stage remains unclear. In this study, 83 participants underwent a 128 electrodes resting-state EEG, a dynamic 100 min tau PET scan (^18^F-MK6240), an amyloid PET scan, a structural T1 MRI scan and neuropsychological assessment. Tau PET data quality control led to a final sample of 66 subjects. Based on the clinical and cognitive status, amyloid and tau PET biomarkers, the group was composed of 37 cognitively unimpaired amyloid negative subjects, 14 cognitively unimpaired amyloid positive subjects and 15 patients with prodromal Alzheimer’s disease. We calculated the average undirected weighted Phase Lag Index in the alpha frequency band with eyes closed and used this as weights for the graph and analysed the global clustering coefficient and characteristic path length in sensor space. As a primary objective, we assessed how these global graph measures correlated with tau PET values, in an *a priori* defined early metaVOI, comprised of the entorhinal and perirhinal cortex, hippocampus, parahippocampus and fusiform cortex. As secondary analyses, we investigated which specific brain regions were mainly implicated, what the contribution was of amyloid, the effect of electrode density and the relation to cognitive performance. In the overall group and within the cognitively unimpaired amyloid positive subgroup, tau aggregation was associated with a decrease in global clustering coefficient and an increase in characteristic path length. These changes reflect the initial disintegration of the small-world brain network during the transitional phase, even before clinical symptoms are apparent. The correlations are most prominent in the perirhinal cortex, indicating that global deterioration of the network is already present early in the Alzheimer’s disease pathology. We obtained similar results with only taking 64 electrodes into account. To conclude, we found that in the asymptomatic stage of Alzheimer’s disease, tau PET load in medial temporal cortex is associated with global electrophysiological measures of network disintegration. The study demonstrates the potential value of high-density EEG in the era of biologically defined Alzheimer’s disease for characterizing brain function in the asymptomatic stage.

## Teaser

Spruyt *et al*. report that in asymptomatic Alzheimer’s disease higher tau PET load in medial temporal cortex is associated with decreasing global clustering coefficient and increasing characteristic path length; graph measures derived from high-density resting-state EEG. This marks the transitional phase between cognitively unimpaired amyloid negative and prodromal Alzheimer’s disease.

## Graphical Abstract

**Graphical Abstract:**
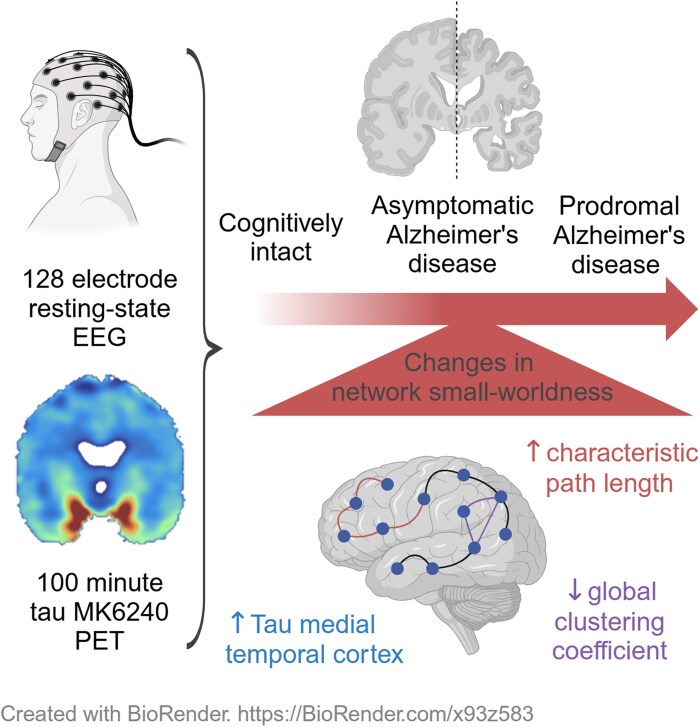

## Introduction

Alzheimer’s disease has a long asymptomatic phase in which pathological changes start decades before the first symptoms appear. Three groups of biomarkers define Alzheimer’s disease: amyloid β (Aβ) deposition, neurofibrillary tangles and biomarkers of neurodegeneration.^1^ These biomarkers together with the cognitive clinical status categorize the disease progression into distinct stages: asymptomatic preclinical Alzheimer’s disease [cognitively unimpaired amyloid positive (CU+)], symptomatic prodromal Alzheimer’s disease (mild cognitive impairment and positive biomarkers) and dementia due to Alzheimer’s disease.^2^ The asymptomatic stage has been further divided into a stable phase of amyloid biomarker positivity (NIA-AA Phase 1) and a phase of within-subject change in cognition (NIA-AA Phase 2).^3^ In the asymptomatic phase, tau PET positivity in the medial temporal cortex has a predictive value for cognitive decline over time,^4^ and marks a transitional phase towards clinical disease expression. This transitional phase of tau aggregation during the asymptomatic stage of Alzheimer’s disease is increasingly used as a target for early drug intervention. How medial temporal tau aggregation during this transitional phase affects brain function remains to be elucidated.

High-density EEG is a promising, non-invasive method to study whole-brain connectivity. This is based on the concept that two time series of neuronal activity are functionally connected when their activities are similar.^2^ Graph theory can be used to quantify the network based on this functional connectivity. This network is a mathematical representation of functional connections (the edges of the network) between all regions (the nodes of the network).^5-7^ For individual estimation of the network based on functional connectivity using graph theory, the most replicable measures are global measures.^8,9^ These graph measures encompass the concept of small-worldness, describing an effective balance between segregation and integration.^6^ This small-worldness is the ratio between the normalized ‘global clustering coefficient’ and the ‘characteristic path length’. The former is a measure of extent to which nodes tend to form clusters,^10^ reflecting the segregation of the network. The latter is an integration measure, quantifying the capacity of a network to become interconnected and to exchange information by measuring the shortest paths connecting pairs of nodes.^5,11^

These graph measures are altered in the clinical stages of Alzheimer’s disease. The clinical stages of Alzheimer’s disease can be also marked as disconnection syndrome, since tau disrupts the axonal microtubule architecture, leading to cognitive impairment and network dysfunction.^12^ How these graph measures change with tau aggregation during the transitional phases of Alzheimer’s disease remains unknown.

Previous studies of functional connectivity in relation to tau have mainly made use of resting-state functional MRI (rsfMRI). These studies revealed that accumulation of tau leads to impaired neuronal connectivity in the symptomatic prodromal stage of Alzheimer’s disease.^13^ Moreover, Schultz *et al.*^14^ report decreased functional connectivity in functional MRI at high concentrations of tau in preclinical Alzheimer’s disease. High-density EEG has been much less frequently combined with tau PET despite EEG’s superior temporal resolution.

Electrophysiologically, clinical Alzheimer’s disease is characterized by a shift in spectral power of the EEG signal, with a reduction in alpha power and an increase in theta power.^2^ The alpha band is the dominant frequency when eyes are closed, is active during general arousal and memory processes,^1^ and is considered to relate to inhibitory neuronal controllers of local circuits.^15^ The oscillatory slowing effect in Alzheimer’s disease was confirmed with magnetoencephalography (MEG).^16^ In addition, a decrease in alpha EEG coherence between electrode pairs has been found in clinical Alzheimer’s disease patients.^11^ In a combined MEG, tau and amyloid PET study, the alpha hyposynchrony colocalized with tau deposition and was modulated by the degree of tau tracer uptake in patients in a prodromal to moderate Alzheimer’s disease dementia stage (Clinical Dementia Rating scale 0.5–2).^17^ In a combined MEG and tau PET study in preclinical and clinical stages of Alzheimer’s disease, functional networks based on amplitude envelope correlations within the alpha and beta frequency band explained the distribution of tau PET tracer better than structural networks did.^18^ In the current study, we investigated the effect of tau aggregation on functional connectivity graph measures of high-density EEG in the alpha band with eyes closed in asymptomatic and prodromal phase of Alzheimer’s disease. As a primary objective, we assessed how tau distribution volume ratio (DVR) values of ^18^F-MK6240 PET in the asymptomatic stage affect global graph measures in the alpha frequency band when eyes were closed. As secondary analyses, we investigated which brain regions were mainly implicated, what the contribution is of Aβ on the effect of tau on these global graph measures, how robust our results are when only taking 64, 32 or 24 electrodes into account, and how this relates to cognitive performance.

## Materials and methods

### Participants

In total, 83 older adults were included in this study: 64 were cognitively unimpaired, and 19 were clinically diagnosed as prodromal Alzheimer’s disease patients (Table 1). The cognitively unimpaired participants were recruited from the community through advertisements in local newspapers for seniors. They were part of the Flemish-Prevent Alzheimer’s disease Cohort KU Leuven (F-PACK), a larger longitudinal study cohort of the Laboratory for Cognitive Neurology, in whom tau PET was also acquired.^19^ One of the F-PACK inclusion criteria is an intact cognitive status based on detailed conventional neuropsychological assessment (see ‘Neuropsychological assessment’ section). Inclusion was stratified genetically according to a factorial design with APOEε4 carrier status (carrier versus noncarrier) as a factor. The stratification at inclusion is such that per protocol, for each 5-year age bin, the APOEε4 carriers and noncarriers are balanced. A detailed description of the full F-PACK inclusion criteria is given in the Supplementary material and also previous reports.^19-21^

**Table 1:** Demographic characteristics and cognitive test scores

	Cognitively unimpaired	Prodromal Alzheimer's disease	Total	Statistics
Participants, *n*	*n* = 64	*n* = 19	*n* = 83	
Age (years)	69.05 ± 8.09 [54; 83]	70.68 ± 6.65 [59; 81]	69.42 ± 7.82 [54; 83]	*T* = −0.89, *P* = 0.38
Sex, male/female	29M/35F	12M/7F	41M/42F	χ^2^ = 2.96; *P* = 0.23
Education (years)	17.16 ± 4.36 [8; 32]	16.21 ± 3.66 [10; 24]	16.94 ± 4.24 [8; 32]	*W* = 695, *P* = 0.35
APOEε4 carriers, *n* (%)	32 (50%)	11 (58%)	43 (52%)	χ^2^ = 2.99; *P* = 0.22
BDNF66 *met* carriers, *n* (%)	29 (45%)	4 (21%)	33 (40%)	χ^2^ = 4.89; *P* = 0.09

Amyloid positive, *n*	*n* = 20	*n* = 19	*n* = 39	
CDR ≥ 0.5, *n*	0	19	19	**χ^2^** **=** **83; *P*** **<** **0.001**
MMSE (/30)	29.20 ± 0.90 [27; 30]	27.21 ± 1.99 [23; 30]	28.75 ± 1.49 [23; 30]	***W*** **=** **999, *P*** **<** **0.001**
AVLT TL (/75)	48.55 ± 9.29 [29; 63]	34.58 ± 9.98 [15; 59]	45.35 ± 11.14 [15; 63]	***W*** **=** **1017, *P*** **<** **0.001**
AVLT DR, %	81.18 ± 17.60 [38; 115]	60.35 ± 32.56 [0; 120]	76.41 ± 23.49 [0; 120]	***T*** **=** **2.67, *P*** **=** **0.01**
BSRT TR (/144)	101.19 ± 19.09 [54; 137]	72.53 ± 19.08 [44; 125]	94.63 ± 22.61 [44; 137]	***T*** **=** **5.74, *P*** **<** **0.001**
BSRT DR (/12)	8.45 ± 2.55 [3; 12]	5.16 ± 2.69 [1; 11]	7.70 ± 2.93 [1; 12]	***W*** **=** **983, *P*** **<** **0.001**
BNT (/60)	54.27 ± 4.17 [40; 60]	47.87 ± 8.88 [24; 57]	52.80 ± 6.18 [24; 60]	***W*** **=** **943, *P*** **<** **0.001**
AVF (no. of words)	20.92 ± 4.57 [8; 33]	15.21 ± 4.54 [7; 23]	19.61 ± 5.16 [7; 33]	***T*** **=** **4.80, *P*** **<** **0.001**
LVF (# words)	37.03 ± 10.91 [9; 59]	31.79 ± 12.20 [18; 67]	35.83 ± 11.42 [9; 67]	***W*** **=** **814. *P*** **=** **0.02**
PALPA49 (/30)	27.19 ± 1.80 [23; 30]	26.37 ± 2.99 [16; 29]	27.00 ± 2.15 [16; 30]	*W* = 704, *P* = 0.29
RPM (/48)	39.63 ± 6.74 [15; 48]	28.11 ± 11.38 [7; 43]	36.99 ± 9.36 [7; 48]	***W*** **=** **1024, *P*** **<** **0.001**
TMT B/A	2.43 ± 0.72 [1.36; 5.42]	2.84 ± 1.62 [1.31; 7.48]	2.52 ± 1.00 [1.31; 7.48]	*W* = 563, *P* = 0.63

Values are presented as mean ± SD [range]. Comparisons between the two subgroups were made using *t*-test, Wilcoxon test or chi-squared test. *P* values corresponding to these comparisons are shown on the right and were indicated in bold if significant (*P* < 0.05). CDR = Clinical Dementia Rating Scale; MMSE = Minimal Mini Mental State Examination; AVLT TL/DR = Auditory Verbal Learning Test Total Learning/Delayed Recall; BSRT TR/DR = Buschke Selective Reminding Test Total Retention/Delayed Recall; BNT = Boston Naming Test; AVF = Animal Verbal Fluency; LVF = Letter Verbal Fluency; PALPA49 = Psycholinguistic Assessment of Language Processing in Aphasia subtest 49; RPM = Raven’s Progressive Matrices; TMT B/A = Trail Making Test ratio B over A.

The patients with prodromal Alzheimer’s disease were recruited in a consecutive series from the Memory Clinic of University Hospital Leuven. Their amyloid biomarker positivity was ascertained during prior clinical work-ups: three patients were positive for Aβ based on Aβ PET and 16 patients were Aβ positive based on CSF. A detailed description of the criteria is given in the Supplementary material.

The Ethics Committee for Clinical Studies UZ/KU Leuven approved the study (S61444), and all participants provided written informed consent for the use of their clinical data for research purposes, in accordance with the Declaration of Helsinki.

### Neuropsychological assessment

All participants were tested with a neuropsychological test battery that covered multiple cognitive domains.^20^ General cognition was analysed with the Clinical Dementia Rating Scale (CDR) and Mini Mental State Examination (MMSE). The Auditory Verbal Learning Test (AVLT) and the Buschke Selective Reminding Test (BSRT) were used to evaluate verbal episodic memory. For the 15-item AVLT, two performance parameters were derived: total learning (AVLT TL), which is the sum of recalled items across all five trials, and delayed recall (AVLT DR), the score of recalled items after 30 min, divided by the score at the fifth trial times 100. From the 12-item BSRT, two parameters were also derived: total retention (BSRT TR), which is the total score of all trials, and delayed recall (BSRT DR), which is the recall score after 30 min. Language and associative-semantic processing were tested with the Boston Naming Test, Animal Verbal Fluency test, Letter Verbal Fluency and the Psycholinguistic Assessment of Language Processing in Aphasia item 49, which tests verbal associative-semantic processing. Executive functioning was analysed with a Trial Making Test and fluid intelligence and reasoning with the standard Raven’s Progressive Matrices (Table 1).

### Image acquisition and processing

#### Structural MRI

Structural MRI data were acquired as coronal slices for all participants on a 3T Philips Achieva system dStream 32-channel headcoil (3D turbo field echo, repetition time (TR)/echo time = 9.6/4.6 ms, flip angle = 8°, TI = 900 ms, voxel size 1 × 1 × 1.2 mm^3^).

#### Amyloid PET

In order to classify the cognitively unimpaired participants in the amyloid negative or amyloid positive subgroup, all cognitively unimpaired participants underwent an amyloid PET scan. Because of tracer availability, we had to switch during the course of the study from a 60 min dynamic ^11^C-Pittsburgh Compound B (PiB) PET (29 subjects) to a static 20 min ^18^F-NAV4694 PET (34 subjects, starting 50 min post-injection). One cognitively intact participant underwent a 20 min ^18^F-florbetaben amyloid PET scan (starting 90 min post-injection) as part of another study in our lab. Details of the acquisition see the Supplementary material. The standard uptake value ratio (SUVR) was calculated for each tracer and converted to Centiloid (CL) (see below).^22^

The details of the procedure for the amyloid PET scans have been described before^19,20^ and are also described in the Supplementary material. Using a standard procedure, a SUVR was derived from the PET scans, using participant-specific cerebellar grey matter as reference region. For the ^11^C-PiB scans, the SUVRs were calculated between 40 and 60 min post-injection, while for the ^18^F-NAV4694 scans this was calculated between 50 and 70 min after the start of the tracer injection and for the ^18^F-florbetaben scans between 90 and 110 min after the start of the tracer injection. The mean SUVR was calculated in a global composite cortical volumes of interest (VOI), which consisted of five bilateral cortical regions, namely frontal (AAL areas 3–10, 13–16 and 23–28), parietal (AAL 57–70), anterior cingulate (AAL 31–32), posterior cingulate (AAL 35–36) and lateral temporal (AAL 81–82 and 85–90).^23,24^

For each tracer, CL values were calculated by converting the SUVR, using the following conversion formula, after validation of the in-house processing procedure:

^11^C-PIB: CL=132.53×SUVR(40−60)−147.64 (Reinartz *et al.*^19^)

^18^F-NAV4694: CL=107.78×SUVR(50−70)−114.71

^18^F-florbetaben: CL=147×SUVR(90−110)−166.5 (De Meyer *et al.*^25^)

The details of the conversion of ^18^F-NAV4694 SUVR to the CL scale are described in the Supplementary Fig. 1. Participants were classified in a binary manner based on a pathologically validated CL cut-off of 23.5 to determine the amyloid positivity.^20^ Based on this classification, cognitively unimpaired participants were assigned to the cognitively unimpaired amyloid negative (CU−) or CU+ subgroup.

#### Tau PET

The primary goal of this study was to investigate the quantitative effect of tau aggregation on EEG based global graph measures. Therefore, all the 83 participants received a 100 min dynamic ^18^F-MK6240 scan on a GE Signa PET/MR scanner with bolus injection in the antecubital vein with a mean injected activity of 151.05 MBq ± 18.43 MBq [87.78–177.58 MBq]. The procedure for tau PET acquisition and analysis are described in detail in the Supplementary material. Images were reconstructed as 30 frames using OSEM (4 iterations and 28 subsets). Quality control involved control of head movement with a maximum of 6 mm translation threshold in any direction and a rotation threshold of 6° in any direction. If a frame exceeded this threshold, the frame was excluded from further analysis. If too many consecutive frames exceeded this threshold, this scan was excluded from further analysis. DVR images with a subject-specific inferior cerebellar mask as reference region were created. Median tau uptake values were calculated in the VOI, which are considered to be the earliest affected regions in the Alzheimer’s disease pathology. The early metaVOI consisted of the entorhinal cortex, perirhinal cortex, caudal and rostral hippocampus, lateral and medial parahippocampus and fusiform cortex and weighted for the number of voxels within each subregion.^26^ Following the criteria of Jack *et al*.,^27^ we also calculated the DVR values in a neocortical VOI, consisting of the weighted average in the inferior and lateral temporal cortex and inferior and medial parietal cortex. Details and Brainnetome labels are shown in the Supplementary material.

#### High-density EEG

In order to relate the EEG derived functional connectivity to tau aggregation, the 83 subjects also underwent a resting-state high-density EEG using 128 active AG/AgCl electrodes placed on a cap following the extended international 10-5 system. The EEG signal was recorded at a sampling rate of 1 kHz using a CE certified Synamps RT amplifier and acquisition software Curry 7 (Compumedics, Australia). For logistic reasons, the EEG was obtained for 35 participants using the actiChamp Plus amplifier and BrainVision Recorder acquisition software (Brain Products GmbH, Germany). There was no significant difference in graph measure outcomes between subjects measured on the two EEG systems. For all subjects, the EEG signal was obtained while resting comfortably in a seated position for 5 min with their eyes open and immediately thereafter for 5 min with their eyes closed. Participants were instructed to fixate on the fixation point at the centre of the screen in the eyes open condition. Details of the procedure are described in the Supplementary material.

The first step of the pre-processing was to identify bad channels and to down-sample the data to 256 Hz, in order to reduce computational load. This was followed by re-referencing to the average of all electrodes. Next, a band-pass filter of [0.5–40 Hz] was applied and independent component analysis was performed to remove automatically detected artefacts, such as blinks and heartbeats. For both the eyes open and eyes closed condition, the first 60 s of the five recorded minutes were discarded, to account for initial accommodation. This resulted in 240 s of data per condition, which was divided into 12 epochs of 20 s. Using the Welch algorithm (with a Hamming filter with size 500 ms), the weighted Phase Lag Index was calculated for all subjects^28-30^ within the alpha frequency band (8–13 Hz) for both the eyes open and eyes closed condition after standard band-pass filtering in EEGlab. These weighted Phase Lag Index values gave rise to an undirected adjacency matrix between electrodes using the absolute value of the weighted Phase Lag Index values as weights. From this adjacency matrix, we calculated the weighted global graph measures in sensor space^31,32^; characteristic path length and global clustering coefficient.^33^

As secondary outcome measures, we also calculated these global graph measures from 24, 32 and 64 electrodes, which were selected from our 128 electrode set-up, based on the 10–10 layout and positions most similar with the caps used in a hospital setting and covering the whole scalp.

In order to verify the well-established shift in power spectra in Alzheimer’s disease in EEG in our sample, we performed a power analysis on our eyes closed resting-state EEG data.

### Statistical analyses

All statistical analyses were conducted with R statistical software version 4.3.2.

#### Primary analyses

The primary objective of this study is to determine how tau aggregation affects global graph measures. A multiple linear regression analysis was performed with, as regressors, tau PET load in the early metaVOI, age, sex and APOEε4 carriership, and, as outcome variable, the global clustering coefficient in the alpha frequency band in the eyes closed condition. A similar analysis was performed with characteristic path length as outcome variable.

If a significant effect was found in the main analysis, an analysis of each of the three subgroups was performed to determine which groups were driving the significant effect. In each of these subgroups, because the assumption of homoscedasticity for a regression analysis was not met in some of the subgroups, a Spearman correlation analysis was performed between tau PET load in the early metaVOI and the two global graph measures. To account for age and sex as covariates, partial correlation analyses were performed.

We also examined whether the global graph measures differed between the three subgroups CU−, CU+ and prodromal Alzheimer’s disease, using a Kruskal–Wallis test and a *post hoc* Dunn’s test.

#### Secondary analyses

To obtain further insight into which regions were mainly driving the effects seen in the main analysis, if a significant effect was found in the primary analysis for the early metaVOI, a secondary analysis was performed where the global graph measures in the alpha frequency band with eyes closed was correlated with tau load in each of the five separate tau-vulnerable regions that constitute the early metaVOI: the entorhinal cortex, perirhinal cortex, hippocampus, parahippocampus and fusiform cortex. This analysis was not corrected for multiple comparisons as it was based on a gatekeeping approach, where a more global analysis is first performed (primary analysis) and more granular analyses are performed subsequently in hierarchical manner when the primary analysis reveals significant results in order to better comprehend the primary effect.

In order to evaluate the effect of amyloid load, we performed a multiple linear regression analysis with the global graph measures as outcome variables, and as regressors global amyloid load, age and sex in the cognitively unimpaired group (*n* = 51). Since we do not have amyloid PET measures of the prodromal Alzheimer’s disease, we restricted this analysis to the CU group.

Since clinical Alzheimer’s disease is typically characterized by a decrease in alpha coherence when the eyes are closed, we focused mainly on this alpha frequency band and the eyes closed condition. For the sake of comparison, we also did an exploratory analysis to assess the effect of tau on global graph measures in the neighbouring theta (4–8 Hz) frequency band and in alpha band eyes open condition.

To assess the robustness of these correlations, we also conducted the analysis with 24, 32 and 64 electrodes. Similar to the primary analysis, we performed a multiple linear regression analysis with, as regressors, tau PET load in the early metaVOI, age, sex and APOEε4 carriership, and, as outcome variable, the global graph measures in the alpha frequency band in the eyes closed condition. If this yielded a significant effect, we next determined the Spearman correlation between the global graph measures and tau PET load in the early metaVOI in each of the subgroups.

In a final secondary analysis, we investigated the association of the global graph measures and tau PET load with neuropsychological performance. These individual factor scores were obtained by performing a factor analysis using the R package *psych* on the following neuropsychological tests: AVLT TL and DR, Buschke TR and DR, Boston Naming Test, Animal Verbal Fluency, Letter Verbal Fluency, Psycholinguistic Assessment of Language Processing in Aphasia 49, Trial Making Test B/A and Raven’s Progressive Matrices. All assumptions to allow performing factor analysis were met: Bartlett’s test for sphericity and correlation adequacy (*P* < 0.001), Kaiser–Meyer–Olking test (>0.06), linearity of pairs of variables and absence of outliers. Factors were rotated with a variance maximizing (varimax) orthogonal rotation, and the goodness of fit of the factor model was assessed using the empirical chi-square statistic Comparative Fit index (>0.90) and the Root Mean Squared Error of Approximation index (<0.10).^20^ To obtain the individual factor scores, factor weights were calculated from the resulting factor loadings, which were used to multiply with the corresponding scaled individual neuropsychological test score (i.e. the Thurstone regression approach).^34^ The correlation between these individual factor scores and the global graph measures and tau PET load was assessed with a partial correlation analysis, with age, sex and education as covariates.

#### Tertiary analyses

We also examined the association between the two global graph measures and tau in the neocortical volume of interest,^27^ in the same way as in the primary analysis for the early metaVOI.

Lastly, we calculated a threshold to determine tau-positivity in the early metaVOI and in the neocortical metaVOI. This threshold was calculated by the mean + 1 SD of 38 CU− participants. Next, we re-divided our total group (*n* = 66) in early metaVOI tau positive and negative; and in neocortical tau positive and negative. Depending on the normality of the distribution of the global graph measures in these two groups, we analysed with a *t*-test or with a Wilcoxon test the difference in global clustering coefficient and characteristic path length between the two groups.

## Results

### Study population

**Figure 1:**
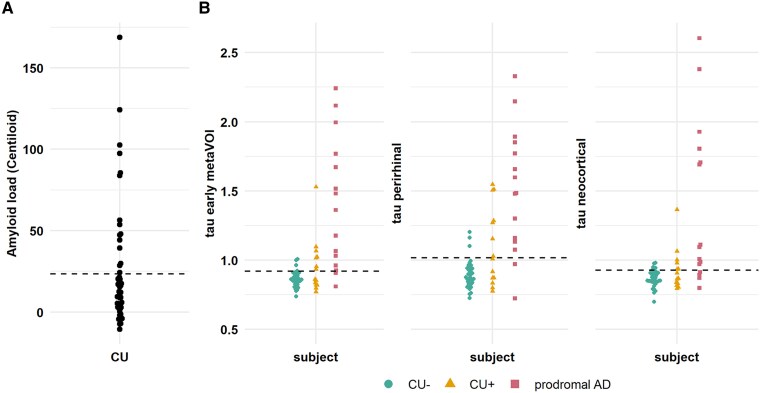
**Amyloid and tau distribution across all subjects.** (**A)** Amyloid CL values of 51 cognitively unimpaired individuals are plotted with a threshold for amyloid positivity of 23.5, indicated with a dashed horizontal line. (**B)** Tau DVR values in early metaVOI, in perirhinal cortex and in neocortical VOI are plotted for all 66 subjects: *n* = 37 CU− subjects shown in green dots, *n* = 14 CU+ subjects shown in orange triangles, *n* = 15 patients with prodromal Alzheimer’s disease shown in red squares. Dashed horizontal line shows the increase tau uptake value in early metaVOI at 0.921, in perirhinal cortex at 1.017 and in neocortical VOI at 0.928. CU− = cognitively unimpaired amyloid negative; CU+ = cognitively unimpaired amyloid positive; prodromal AD = prodromal Alzheimer’s disease; PET DVR = positron emission tomography distribution volume ratio.

In total, 83 participants underwent neuropsychological assessment, a 128 electrodes resting-state EEG, a dynamic tau PET scan (^18^F-MK6240), an amyloid PET scan (^18^F-NAV4694, ^11^C-PiB or a ^18^F-florbetaben) and a structural T1 MRI scan. Based on a CL cut-off of 23.5, cognitively unimpaired subjects were classified as amyloid negative or positive. This resulted in 44 CU− subjects and 20 CU+ subjects, along with the 19 patients with prodromal Alzheimer’s disease. Figure 1A shows the amyloid distribution across all cognitively unimpaired subjects.

Based on the *a priori* thresholds set for acceptable ranges of movement (see Materials and methods section) during the 100 min ^18^F-MK6240 PET scan, we had to exclude 17 participants for the analyses with neurofibrillary tangles, out of which 7 were CU−, 6 were CU+ and 4 with prodromal Alzheimer’s disease. There were no differences between the included and excluded subjects across all demographic variables. This created a new subset of 66 subjects in total, out of which 37 were categorized as CU− (mean age = 66.84 ± 7.0), 14 were CU+ (mean age = 74.21 ± 6.97) and 15 patients with prodromal Alzheimer’s disease (mean age = 71.4 ± 6.5) (Table 2). The three groups did not differ in gender or educational level. There was a significant difference for age between the CU− and CU+ subgroups (*z* = −3.05, *P* = 0.003).

**Table 2:** Demographic characteristics of subset of 66 subjects with group differences

	CU−	CU+	Prodromal Alzheimer's disease	Total	Statistics
Participants, *n*	*n* = 37	*n* = 14	*n* = 15	*n* = 66	
Age (years)	66.8 ± 7.0 [54, 81]	74.2 ± 7.0 [64, 83]	71.4 ± 6.5 [60, 81]	69.4 ± 7.6 [54, 83]	**χ^2^** **=** **10.40** ***P*** **=** **0.006**
Sex, male/female	15M/22F	7M/7F	10M/5F	32M/34F	χ^2^ = 2.89 *P* = 0.24
Education (years)	17.2 ± 5.0 [8, 32]	16.4 ± 2.7 [12, 22]	16.3 ± 3.1 [10, 23]	16.8 ± 4.2 [8, 32]	χ^2^ = 0.28 *P* = 0.87
*APOEε4*, *n* (%)	17 (45.9%)	11 (78.6%)	8 (53%)	36 (55%)	χ^2^ = 4.31 *P* = 0.12
BDNF66*met*, *n* (%)	18 (48.6%)	3 (21.4%)	3 (20%)	24 (36%)	χ^2^ 5.42, *P* = 0.07
Tau early metaVOI	0.86 ± 0.06 [0.74, 1.01]	0.96 ± 0.19 [0.77, 1.53]	1.40 ± 0.47 [0.81, 2.24]	1.00 ± 0.33 [0.74, 2.24]	**χ^2^** **=** **22.44** ***P*** **<** **0.001**
Tau entorhinal	0.73 ± 0.08 [0.58, 0.88]	0.88 ± 0.25 [0.55, 1.51]	1.03 ± 0.30 [0.59, 1.69]	0.83 ± 0.23 [0.55, 1.69]	**χ^2^** **=** **15.86** ***P*** **<** **0.001**
Tau perirhinal	0.89 ± 0.10 [0.73, 1.20]	1.10 ± 0.27 [0.78, 1.55]	1.50 ± 0.45 [0.72, 2.33]	1.08 ± 0.36 [0.73, 2.33]	**χ^2^** **=** **23.42** ***P*** **<** **0.001**

Values are presented as mean ± SD [range]. Comparisons between three subgroups were made using Kruskall–Wallis tests. *P* values corresponding to these comparisons are shown on the right and were indicated in bold if significant (*P* < 0.05). CU− and CU+ = cognitively unimpaired amyloid negative and positive, respectively.

There was a significant difference in tau PET load in the early metaVOI, between CU− and prodromal Alzheimer’s disease and between CU+ and prodromal Alzheimer’s disease (both *p*_adj_ < 0.001), but not between CU− and CU+. Additionally, there was a significant difference in tau PET load in the entorhinal cortex and perirhinal cortex between CU− and prodromal Alzheimer’s disease (both *p*_adj_ < 0.001), in the perirhinal cortex between CU+ and prodromal Alzheimer’s disease (*p*_adj_ = 0.044), but not between CU− and CU+ (Table 2 and Supplementary Table 1). Figure 1B shows the tau DVR distribution in the early metaVOI as well as in the perirhinal cortex and neocortical VOI.

In the eyes closed resting-state EEG data, we confirmed the typical ‘slowing effect’ as the shift in spectral power, with relatively less alpha power and more theta power in the prodromal Alzheimer’s disease group, compared to the CU groups (Supplementary Fig. 2).

### Primary outcome analyses

In the whole group multiple linear regression analyses, the model was significant for both the clustering coefficient (*P* = 0.0098) and for the characteristic path length (*P* = 0.04). In the regression models, tau PET load in the early metaVOI was significantly associated with a decrease in global clustering coefficient and an increase in characteristic path length, respectively. None of the other regressors had a significant effect (Table 3).

**Table 3:** Relationship between the global graph measures in the alpha frequency band with eyes closed and tau PET load in early metaVOI in whole group

	Global clustering coefficient		Characteristic path length	
Model	*β*	*P*	*β*	*P*
**Tau in early metaVOI**	−0.02	**0**.**003**	1.40	**0**.**004**
Age	0.005	0.40	0.43	0.38
Sex	0.02	0.16	−0.69	0.47
APOEε4	−0.004	0.72	−0.45	0.63

Regression model with global graph measures in the alpha frequency band in the eyes closed condition as dependent variables and tau PET load in the early metaVOI, age, sex and APOEε4 carriership as independent variables. Both the regression coefficient *β* and *P* value are shown and were indicated in bold if significant (*P* < 0.05). *n*  *=* 66.

**Figure 2:**
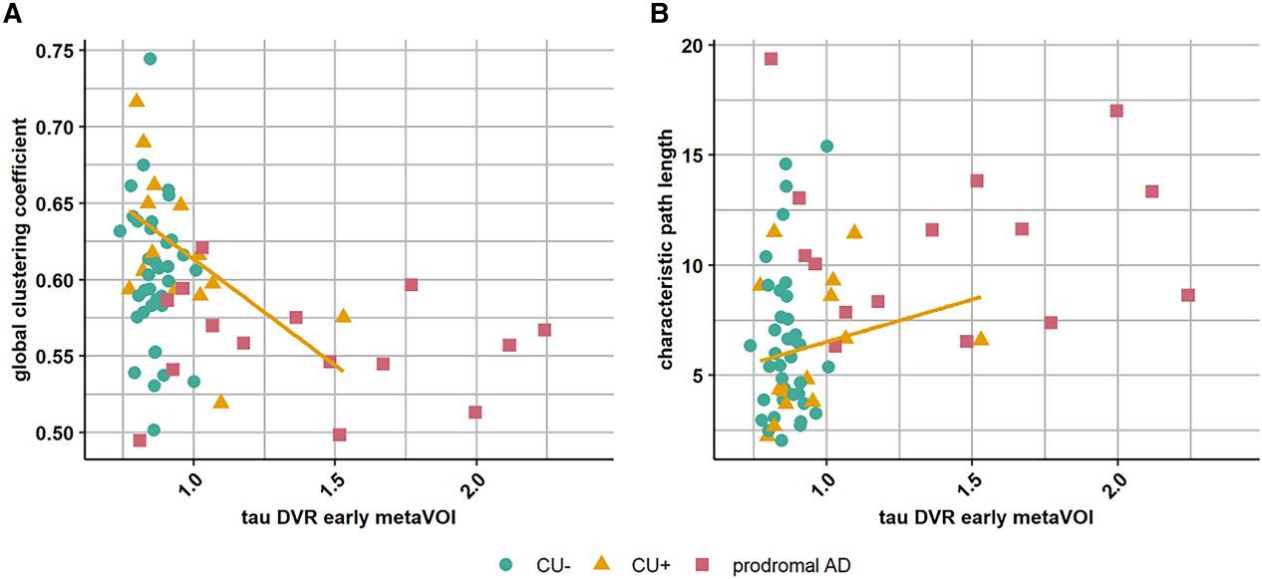
**Correlation between tau PET DVR in early metaVOI and global graph measures. (A)** Correlation between tau in early metaVOI and the global clustering coefficient, (**B**) and the characteristic path length. *n* = 37 CU− subjects shown in dots, *n* = 14 CU+ subjects shown in triangles, *n* = 15 patients with prodromal Alzheimer’s disease shown in squares. Regression line in orange is shown for the CU+ subgroup. Statistical analyses were performed in the total group (*n* = 66) using multiple linear regression analyses, in which tau PET load in the early metaVOI was significantly associated with a decrease in global clustering coefficient and an increase in characteristic path length. Statistical analyses in the subgroups was performed using Spearman correlations, with the following correlation coefficients in the CU+ group: *ρ* = −0.62 for global clustering coefficient, *ρ* = 0.31 for characteristic path length. *P* value was *P* = 0.02 for global clustering coefficient and *P* = 0.29 for characteristic path length. CU− = cognitively unimpaired amyloid negative; CU+ = cognitively unimpaired amyloid positive; prodromal AD = prodromal Alzheimer’s disease; PET DVR = positron emission tomography distribution volume ratio.

Next, we investigated the correlation between the two global graph measures and tau PET load in the early metaVOI within each of the three subgroups separately (CU−, CU+ and prodromal Alzheimer’s disease). In the CU+ subgroup, there was a significant negative correlation between the global clustering coefficient and tau PET load in early metaVOI, which became borderline significant when we corrected for age and sex as covariates (*ρ* = −0.62, *P* = 0.02, *p*_adj_ = 0.05) (Fig. 2). The correlation between the characteristic path length and tau PET load in the early metaVOI was not significant. There were no significant correlations in the CU− subgroup, nor in the prodromal Alzheimer’s disease subgroup (Table 4).

**Table 4:** Relationship between tau load in early metaVOI and the global graph measures in the alpha frequency band with eyes closed

	Global clustering coefficient and tau early metaVOI				Characteristic path length and tau early metaVOI			
	*ρ*	95% CI	*P*	covariates	*ρ*	95% CI	*P*	covariates
CU−	−0.12	−0.45 to 0.18	0.48	*ρ* = −0.11 ; *P* = 0.53 95% CI = −0.43 to 0.24	−0.05	−0.36 to 0.28	0.78	ρ = −0.07 ; *P* = 0.70 95% CI = −0.37 to 0.28
CU+	−0.62	−0.86 to −0.13	**0**.**02**	*ρ* = −0.57 ; *P* = 0.05 95% CI = −0.87 to 0.04	0.31	−0.27 to 0.72	0.29	ρ = 0.34 ; *P* = 0.27 95% CI = −0.32 to 0.78
Prodromal Alzheimer's disease	−0.08	−0.57 to 0.45	0.77	*ρ* = −0.29 ; *P* = 0.34 95% CI = −0.74 to 0.34	−0.004	−0.51 to 0.51	0.99	ρ = 0.10 ; *P* = 0.73 95% CI = −0.50 to 0.64

Spearman ρ correlations between tau PET in early metaVOI and global graph measures in the three subgroups separately. The left column indicates each time the Spearman correlation coefficient, followed by the 95% confidence interval and the *P* value, the right column indicates the partial correlation coefficients *ρ*, *P* values and 95% confidence interval after adjusting for age and sex. *P* values were indicated in bold if significant (*P* < 0.05).

The average global graph measures per subgroup are shown in Table 5. The clustering coefficient and the characteristic path length differed significantly between the CU− versus the prodromal Alzheimer’s disease subgroup (clustering coefficient: *z* = −3.15, *p*_adj_ = 0.002; path length: *z* = −3.81, *p*_adj_ < 0.001), as well as between the CU+ versus the prodromal Alzheimer’s disease group (clustering coefficient: *z* = 3.62, *p*_adj_ < 0.001; path length: *z* = 3.45, *p*_adj_ < 0.001). There was no significant difference between the CU− and CU+ subgroups (Supplementary Table 2).

**Table 5:** Average global graph measures per subgroup with group differences

	CU−	CU+	Prodromal Alzheimer's disease	Total	Statistics
Participants, *n*	*n* = 44	*n* = 20	*n* = 19	*n* = 83	
Global clustering coefficient	0.60 ± 0.05 [0.50, 0.74]	0.61 ± 0.05 [0.52, 0.72]	0.56 ± 0.04 [0.49, 0.62]	0.59 ± 0.05 [0.49, 0.74]	**χ^2^** **=** **14.63** ***P*** **<** **0.001**
Characteristic path length	6.57 ± 3.29 [2.05, 15.42]	6.37 ± 3.04 [2.23, 12.72]	10.51 ± 3.69 [4.90, 19.38]	7.42 ± 3.73 [2.05, 19.38]	**χ^2^** **=** **16.66** ***P*** **<** **0.001**

Values are presented as mean ± SD [range]. Comparisons between three subgroups were made using Kruskall–Wallis χ^2^ tests. *P* values corresponding to these comparisons are shown on the right and were indicated in bold if significant (*P* < 0.05). CU− and CU+ = cognitively unimpaired amyloid negative and positive, respectively.

### Secondary outcome analyses

In each of the five separate tau-vulnerable regions that constitute the early metaVOI, except for the entorhinal cortex, the whole group multiple linear regression models were significant for both the clustering coefficient and for the characteristic path length as outcome variables. In the regression models, the effect of tau PET load in each region was significantly associated with a decrease in global clustering coefficient and an increase in characteristic path length, respectively. None of the other regressors (age, sex and APOEε4 carriership) had a significant effect (Supplementary Table 3).

**Figure 3:**
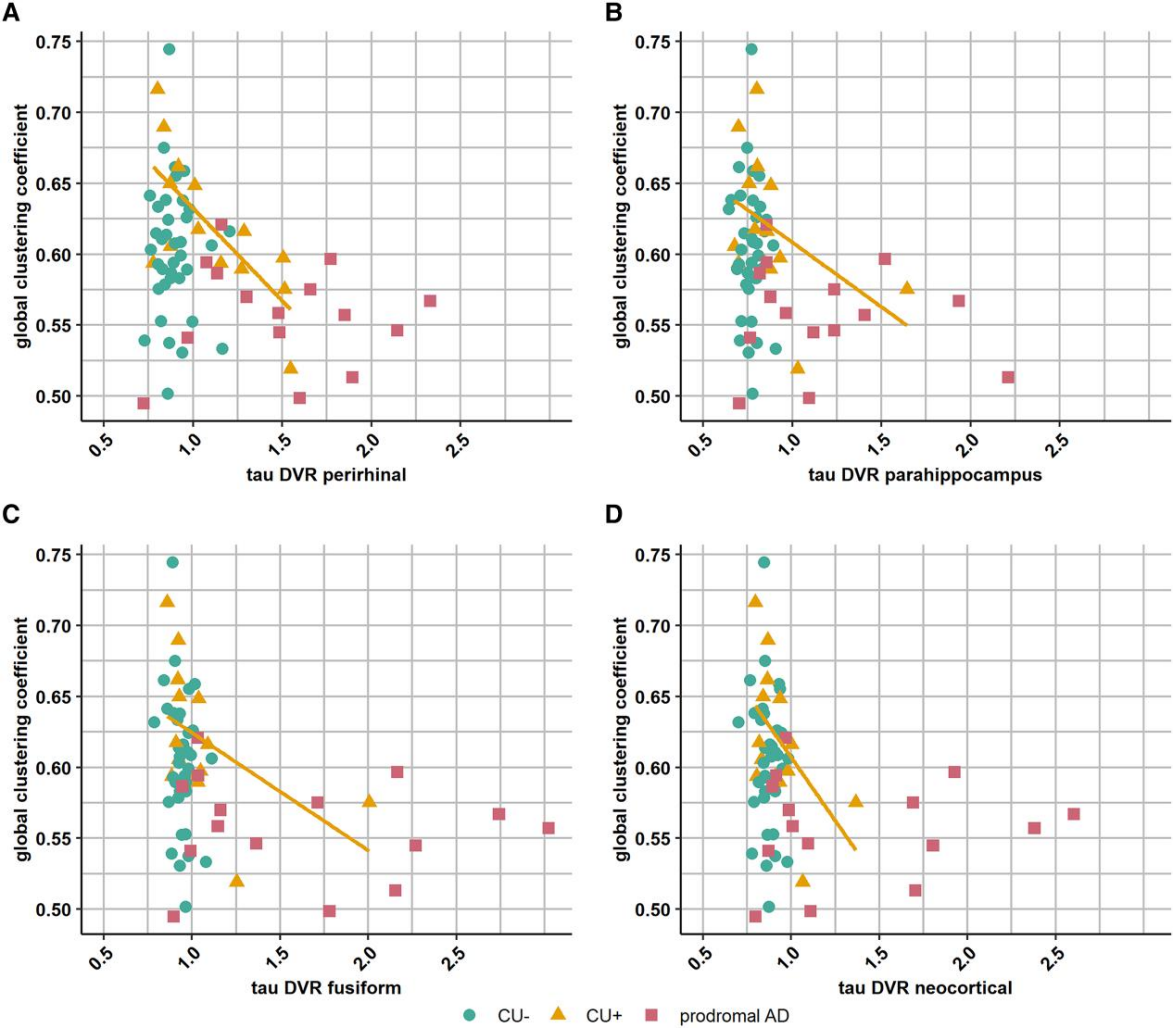
**Correlation between the global clustering coefficient and tau PET DVR in perirhinal cortex, parahippocampus, fusiform and neocortical VOI.** (**A**) Spearman correlation between the global clustering coefficient and tau in the perirhinal cortex, (**B**) tau in parahippocampus, (**C**) tau in fusiform, (**D**) tau in neocortical VOI. *n* = 37 CU− subjects shown in dots, *n* = 14 CU+ subjects shown in triangles, *n* = 15 patients with prodromal Alzheimer’s disease shown in squares. Regression line in orange is shown for the CU+ subgroup. Statistical analyses were performed in the subgroups using Spearman correlations, with the following correlation coefficients in the CU+ group: *ρ* = −0.69 for perirhinal cortex, *ρ* = −0.55 for parahippocampus, *ρ* = −0.63 for fusiform and *ρ* = −0.56 for neocortical VOI. FDR corrected *P* values were significant (*p*_adj_ < 0.05) for all regions, except for parahippocampus (*P* = 0.04; *p*_adj_ = 0.16). CU− = cognitively unimpaired amyloid negative; CU+ = cognitively unimpaired amyloid positive; prodromal AD = prodromal Alzheimer’s disease; PET DVR = positron emission tomography distribution volume ratio.

When we analysed the subgroups separately, the CU+ subgroup showed a significant correlation between the global clustering coefficient and tau PET load in the perirhinal cortex (*ρ* = −0.69; *p*_uncor_ = 0.008; *p*_adj_ = 0.01) and fusiform cortex (*ρ* = −0.63; *P* = 0.019; *p*_adj_ = 0.04) after adjusting for age and sex (Table 6 and Fig. 3). There were no significant correlations in the CU− subgroup, or in the prodromal Alzheimer’s disease subgroup (Supplementary Table 4).

**Table 6:** Relationship between tau load in different regions and the global graph measures in the alpha frequency band with eyes closed in the CU+ group

Global clustering coefficient				
	*ρ*	95% CI	*p*_uncor_	covariates
Entorhinal	−0.50	−0.81 to 0.05	0.07	*ρ* = −0.42; *P* = 0.17; 95% CI = −0.79 to 0.17
Perirhinal	−0.69	−0.89 to −0.25	**0**.**008**	*ρ* = −0.69; ***P*** **=** **0.01;** 95% CI = −0.90 to −0.23
Hippocampus	−0.30	−0.71 to 0.28	0.30	*ρ* = −0.37; *P* = 0.24; 95% CI = −0.76 to 0.23
Parahippocampus	−0.55	−0.84 to −0.03	**0**.**04**	*ρ* = −0.43; *P* = 0.16; 95% CI = −0.80 to 0.15
Fusiform	−0.63	−0.87 to −0.14	**0**.**019**	*ρ* = −0.60; ***P*** **=** **0.04;** 95% CI = −0.87 to −0.08

Spearman ρ correlations between tau PET in the different regions and global graph measures in the CU+ subgroup. The left column indicates the Spearman correlation coefficient, followed by the 95% confidence interval and the uncorrected *P* value, the right column indicates the partial correlation coefficients *ρ*, uncorrected *P* values and 95% confidence interval after adjusting for age and sex. *P* values were indicated in bold if significant (*P* < 0.05). *n* = 14.

As a further secondary analysis in the cognitively unimpaired group (*n* = 51), we applied a multiple linear regression model with the two global graph measures as outcome variables, and, as regressors, the amyloid PET CL score, age and sex. The overall model for the global clustering coefficient was significant (*P* = 0.001), and borderline significant for the characteristic path length (*P* = 0.049). None of the other regressors had a significant effect, except for sex (clustering coefficient: *β*_sex_ = 0.04, *p*_sex_ = 0.002; path length: *β*_sex_ = −2.43, *p*_sex_ = 0.01). Results of the independent variables are shown in Supplementary Table 5.

For the sake of comparison, we also did an exploratory analysis with tau load and global graph measures in the theta frequency band and the alpha frequency eyes open condition. There were no significant effects (Supplementary Table 6).

When only taking 64 electrodes into account, we found similar results for both the whole group analysis and in the separate subgroups, compared with 128 electrodes (Supplementary Tables 7 and 8). When only taking 32 or 24 electrodes into account, only the whole group regression analysis was significant (Supplementary Table 7).

To assess if the changes in global graph measures have implications on neuropsychological performance levels, we investigated the association of the global graph measures and tau PET load with the individual factor scores from the neuropsychological assessment.

Conditions for performing a factor analysis were met: the Bartlett’s test of sphericity had a *P* value of 4.07e^−57^, the Kaiser–Meyer–Olking test for sampling adequacy had a value of 0.83 (>0.6), and there were no outliers in the data of the neuropsychological test scores. The model fit parameters were also within the recommended norms: an empirical chi-square of 15.27 (*P* < 0.95), comparative fit index of 0.99 (>0.9) and a root mean square error of approximation index of 0.072 (<0.1). This gave rise to two factors, which explained 50.5% of the total variance: a first factor with an eigenvalue of 2.91 had the BSRT TR test as the highest factor loading with 0.88, and a second factor with an eigenvalue of 2.14 had Boston Naming Test as the highest factor loading with 0.68 (Supplementary Table 9). These results are in line with a previous study of Schaeverbeke *et al*.^20^

Next, we investigated the association of the individual factor scores and the global graph measures and tau PET load, respectively. As expected,^20,35^ there was a significant correlation between the individual factor scores from the first factor reflecting episodic memory performance adjusted for age, sex and education, and the tau PET load in the early metaVOI in the whole group analysis (*n* = 66) (*ρ* = −0.33, *P* = 0.008). There were no significant correlations found in the subgroups separately. Individual factor scores did not correlate with global graph measures (except for a borderline correlation in the whole group analysis (*n* = 83) between the individual factor scores from the second factor and the characteristic path length (*ρ* = −0.32, *p*_uncorrected_ = 0.01), when adjusted for age, sex and education).

### Tertiary outcome analyses

We also examined the associations between the two global graph measures and tau in the neocortical volume of interest.^27^ In the whole group regression analysis, the overall model was not significant for the global clustering coefficient (*P* = 0.08), nor for the characteristic path length (*P* = 0.12).

Lastly, we calculated a threshold to determine tau-positivity in the early metaVOI, in the different regions separately and in the neocortical metaVOI. Based on this threshold, we re-divided our total group (*n* = 66) in early metaVOI tau positive (*n* = 24) or negative *(n* = 42). There was a significant difference in global clustering coefficient (*t* = 2.99; *P* = 0.004) and characteristic path length (*W* = 326; *P* = 0.017) between the two groups. When we re-divided the total group (*n* = 66) in neocortical tau positive (*n* = 23) or negative (*n* = 43), we did not find differences in global clustering coefficient or characteristic path length between these two groups.

## Discussion

Tau aggregation in the asymptomatic stage of Alzheimer’s disease marks a transitional phase between stable asymptomatic amyloid positivity and the clinically manifest stage. The current multimodal study reveals that, in this transitional phase, aggregated tau in medial temporal cortex is associated with a disruption of global network connectivity: the amount of medial temporal tau PET tracer retention is associated with a decrease in global clustering coefficient and an increase in characteristic path length. These changes reflect the initial disintegration of the small-world brain network during this transitional phase and provide evidence for the negative impact of tau aggregates at a neurobiological level even prior to symptom onset.

The statistical analysis determines the association between tau PET load and the graph measures both across the entire group and in the separate subgroups. Syndrome diagnosis covaries with tau PET load, as all cases with prodromal Alzheimer’s disease and some cognitively intact cases with amyloid positivity have an increase in tau PET load. It is therefore important to consider in the whole group analysis an indirect effect mediated through syndrome. However, the correlation is strongest within the subgroup containing cases with both low and high tau PET levels (Table 4). This is a strong argument for a direct relationship rather than an purely indirect effect due to between-group differences. If the association were indirect and due to the effect of syndrome, we would not expect that within one of the three subgroups there would be a significant correlation between tau PET load and global clustering coefficient. Furthermore, the association is strongest in the region that is affected earliest in the Alzheimer’s disease, the perirhinal cortex, which also strengthens this argument (Table 6). We therefore strongly argue that the data are not indicative of a spurious indirect effect but reflect the association between tau PET and global clustering coefficient in the early asymptomatic disease stage.

When considering the subgroups separately, the associations between tau aggregation and global graph measures was almost exclusively driven by the subgroup of amyloid positive cognitively normal individuals. This points to the critical nature of this transitional phase in the disease process characterized by medial temporal tau aggregation as well as functional changes in electrophysiological network dynamics. The numerically highest correlation with the global clustering coefficient was obtained with perirhinal tau levels (Table 6). The perirhinal cortex in the Brainnetome atlas corresponds to Brodman area 35 and 36. It is well established that this is one of the initial regions of predilection for tau aggregation neuropathologically,^36^ as well as on tau PET.^37^ The current data indicate that perirhinal tau accumulation leads to distributed changes in network connectivity, possibly through its impact on the long-range anatomical connections from perirhinal cortex to the neocortical association regions. Another possibility is that the increase in tau aggregation in perirhinal cortex impacts on the afferent input to the hippocampus and that the global connectivity changes are mediated through the effect on the hippocampus. The network disruption could also theoretically relate to medial temporal hyperexcitability that may be associated with the tau aggregation in the hippocampal formation,^38,39^ or be due to dysfunction of critical medial temporal electrophysiological hubs. Alternatively, increased perirhinal tau levels are also associated with more widespread tau aggregation and the disruption of global connectivity may result from local effects of more widespread tau aggregation in early affected neocortical regions.^40^ In this study, we also found a weak correlation between global graph measures and tau PET load in the neocortical VOI. A contribution of amyloid accumulation independent of tau cannot be excluded, however in our dataset we did not find any positive evidence for such an association between amyloid load and global graph measures.

In the amyloid negative group, values for the two global graph measures exhibited a wide spread. The wide variability in the amyloid negative healthy controls was not related to tau levels or neuropsychological scores. There may be other explanatory factors that remain to be determined in amyloid negative cognitively intact individuals. Moguilne *et al.*^41^ report accelerated brain aging influenced by sex, geographical, macrosocial and disease-based factors. In any case, given this wide variability in the amyloid negative cognitively intact subjects and the substantial overlap with the values in the amyloid positive cases, the global graph measures are unlikely to be a suitable Alzheimer’s disease biomarker in the asymptomatic stage for individual diagnostic purposes. The potential value of EEG based network measures would lie more in the group-based characterization of brain network function during the transitional phase, or possibly in longitudinal analysis within subjects, but this needs to be further investigated.

At the other ‘extreme’ of the spectrum, in the prodromal Alzheimer’s disease group, the global clustering coefficient was decreased and the characteristic path length was increased compared to the two cognitively intact groups. The absence of a correlation with tau load in the prodromal Alzheimer’s disease group may indicate that once tau aggregation is spread beyond the early affected regions, the disruption of connectivity has reached a lower level that does not vary much within a same global disease stage (i.e. NIA-AA stage 3 in the current study). However in the current dataset, there was also a trend between the neocortical, and not only the medial temporal, tau burden and the global clustering coefficient in the amyloid positively cognitively intact controls. Conceivably, inclusion of a wider range of symptomatic and biological stages would yield a correlation between tau aggregation levels and global network characteristics also in the symptomatic group. Alternatively, in the mild and later dementia stages, disrupted connectivity may relate to other factors than tau burden, such as structural neurodegeneration. Since the current analysis is sensor-based, it remains speculative to which degree the changes in EEG connectivity originate from medial temporal changes or from the convexity or medial surface of the hemisphere. Studies have shown that it is possible to capture sources from the mediotemporal lobe using high-density EEG.^31,32^

Several studies report changes in the small-worldness in clinical Alzheimer’s disease, resulting in an imbalance between local connectedness and global integration of a network.^2,42,43^ Comparing patients with mild to moderate Alzheimer’s disease with controls, De Haan *et al.*^44^ report a decrease in global clustering coefficient and a decrease in characteristic path length in the alpha band with low-density EEG, while Cecchetti *et al.*^45^ report a decrease in clustering coefficient and an increase in path length in the alpha frequency band. The current study confirms this change in small-worldness since we also find a decrease in global clustering coefficient and an increase in characteristic path length. This leads to less efficient information exchange across brain areas and is in line with the disconnection hypothesis of Alzheimer’s disease.^5^ It is worth noting that both increases and decreases in path length may signify disruption of connectivity and loss of structure of a network. Regarding the graph measures global clustering coefficient and characteristic path length in clinical Alzheimer’s disease, there are many inconsistencies in literature, related to methodological differences.^6^ Different electrophysiological studies have different subject populations (age, comorbidities, Mini Mental State Examination scores and Alzheimer’s disease stage, sample sizes), have different ways of (pre)processing, use different functional connectivity measures, or use different EEG properties (amplitude, phase, frequency). Furthermore, neurodegeneration is a nonlinear process, that can induce distinct conditions in different stages of the pathology.^46^ A strength of the current multimodal approach is the simultaneous acquisition of high-density EEG and a robust biological measure of aggregated tau. The biological characterization of Alzheimer’s disease may help to resolve some of the ambiguities present in EEG studies based on mainly clinical characterizations.

The current high-density EEG findings are also in line with MEG findings of a loss of synchrony and coherence within the alpha band.^17^ A previous MEG study in clinical Alzheimer’s disease in a more advanced clinical stage also reported a decrease in global clustering coefficient and an increase in path length in the lower alpha band,^47^ in line with our EEG findings. However, after normalization for network size, the direction of the change in path length changed to a decrease.^47^

Numerous studies have evaluated the link between how tau aggregation is distributed across regions and the interregional connectivity based on rsfMRI.^37,48-50^ In a group consisting of cognitively intact subjects with or without amyloid positivity and amyloid positive mild cognitive impairment patients, tau deposition in transentorhinal cortex (which corresponds to perirhinal cortex) is associated with reduced rsfMRI connectivity between Brodman area 35 and networks including orbitofrontal/medial prefrontal cortex, as well as posterior hippocampus, parahippocampal gyrus fand angular gyrus. This tau-related reduced rsfMRI connectivity in cognitively unimpaired individuals is associated with early subtle memory impairment.^37^ Hrybouski *et al*.^40^ describe a phase of increased rsfMRI connectivity between anterior medial temporal regions in cognitively intact, amyloid positive subjects that is not present in the prodromal Alzheimer’s disease patients. Likewise, Schultz *et al*.^14^ describe increased rsfMRI connectivity within the default mode network and the salience network in amyloid positive individuals with low tau levels (hyperconnectivity phase) and decreased connectivity in amyloid positive cognitively intact individuals with higher tau levels (hypoconnectivity phase). The correlation between tau PET levels and global graph measures in our study, particularly in the cognitively intact amyloid positive group, may result from a combination of stronger connectivity in the earliest tau aggregation phase in some cases and weaker connectivity in cases with higher tau load. This may be in line with these earlier fMRI-based reports.^39^ It is worth noting that even these high and low graph connectivity measures still fall within the range observed in the cognitively intact amyloid negative cases (Figs 2 and 3), the main distinction being that a significant portion of the variance in global graph measures can be explained by the tau levels only in the amyloid positive cognitively intact individuals. Rossini *et al*.^2^ reported that dementia pathology mainly affects synaptic transmission in the very early stages. These synaptic transmission changes can be detected with EEG, reflecting the summation of excitatory and inhibitory oscillatory post-synaptic potentials from pyramidal neurons, and it is therefore able to detect synchronization of the activity in the same directions.^42,51,52^ Ferreri *et al.*^5^ reported that functional connectivity alterations affecting this synaptic dysfunction precede structural alterations in the brain, which might explain our findings in the CU+ subgroup.

We only found effects in the alpha frequency band when eyes were closed and did not find any significant correlations between tau PET load and graph measures in the theta band or in the eyes open condition. This is in line with the previous studies that also report changes in PLI values or graph measures limited to the alpha frequency.^11,53^ Ranasinghe *et al.*^54^ state with MEG that excitatory neuronal parameters in the alpha band are associated with increased tau depositions in patients with Alzheimer’s disease, concluding that a net reduction of alpha represents dysregulated network activity.^54^

We found a significant correlation between neuropsychological factor scores and tau in the early metaVOI across the entire group, but not with the global graph measures. It is well established that an increase of tau is associated with cognitive change.^35,37^ In the current study, the global graph measures correlated with tau load but not with cognitive performance. Possibly, the changes in graph-based connectivity may partly reflect compensatory mechanisms which would reduce the probability of finding correlation with the conventional cognitive assessment scores. Another explanation might be that the cognitive tests developed for clinical diagnosis are not sensitive enough to capture the changes in global graph measures that precede the clinical stages.

The current data point to the potential value of high-density EEG in the era of biologically defined Alzheimer’s disease to characterize the functional impact of tau aggregation in the asymptomatic phase. Potential contexts of use to be further explored are its use as a marker for the effect of interventions on brain function in the transitional phase, e.g. longitudinally, or as a stage marker of preclinical changes of brain network function. More longitudinal research is needed for further validation.

The strengths of this study are the well-defined community-recruited F-PACK cohort, which is independent from other studies and who underwent extensive neuropsychological assessments covering multiple cognitive domains as well as ^18^F-MK6240 PET scans, amyloid PET scans, and a high-density EEG experiment. Moreover, this cohort was enriched for genetic Alzheimer’s disease risk factors (APOEε4 and BDNF*val66met* carriers), promoting the CU+ participants to convert to prodromal Alzheimer’s disease.^12^ However, there are also some limitations to this study. Collecting DVR data from a 100-min tau PET scan, 128-electrode high-density EEG, amyloid PET scan, MRI scan and neuropsychological testing is highly challenging, in particular for the participant. As a consequence and because of strict a priori quality control criteria, we had to exclude 17 participants to maintain and ensure high quality data for the long dynamic tau PET acquisition. We analysed the graph measures in sensor space, with the drawback of volume condition affecting spatial resolution.^10^ Additionally, statistical interdependencies of the functional connectivity measures may be biased by the effect of volume conduction, and by the influence of the reference electrode and confounding factors, such as biological and instrumental noise.^11,52^ However, since we found similar results for caps with a 64 electrodes, we argue that the results and conclusions are not significantly impacted by these effects of volume conduction. Also, a more liberal cut-off for amyloid binarization could be applied. We used a cut-off CL value of 23.5, based on Reinartz *et al*.,^19^ but using thresholds is always somewhat ambiguous since true positives who have underlying Alzheimer’s disease pathology may be unjustly removed, while false positives or participants with Aβ due to other pathology such as DLB may sometimes be included.^55^ A last limitation is that this study is cross-sectional. Additional longitudinal research is needed to better understand the longitudinal changes of the Alzheimer’s disease pathology and the effects of tau on graph measures. The current analysis is performed in sensor space and limited to two global graph measures. The restricted number of outcome parameters allowed us to keep the numbers of comparison under control. In the future, source reconstructed analysis may allow for analyses directed at specific regions or networks.

To conclude, we found that in the asymptomatic stage of Alzheimer’s disease, focal tau PET load in medial temporal cortex is associated with global electrophysiological measures of network disintegration. Tau aggregation and network disintegration mark the transitional phase between cognitive unimpaired amyloid negative normal functional connectivity on the one hand, and prodromal Alzheimer’s disease on the other hand. The current data show the potential value of high-density EEG for characterizing brain network function in this transitional phase.

## Supplementary Material

fcaf138_Supplementary_Data

## Data Availability

The data that support the findings of this study are available from the corresponding author upon reasonable request.
