# Supplementary material for: EEG-based graph network analysis in relation to regional tau in asymptomatic Alzheimer’s disease
Source: Brain Commun. 2025 Apr 15;7(2):fcaf138. doi: 10.1093/braincomms/fcaf138 (PMC12008720; doi:10.1093/braincomms/fcaf138)
Supplement: fcaf138_Supplementary_Data [file fcaf138_supplementary_data.docx]

# Supplementary material

## Supplementary methods

### **Study participants**

All subjects are part of wave 5 of the Flemish-Prevent AD Cohort KU Leuven (F-PACK). The inclusion criteria for the cognitively intact participants were age 50-80 years, a Clinical Dementia Rating Scale (CDR) of 0, a Mini Mental State Examination (MMSE) ≥ 27, and having to score within published norms on a standard neuropsychological examination. Inclusion was stratified genetically according to a factorial design, with two factors: APOEε4 carrier status (carrier versus noncarrier) and BDNF *val66met* carrier status (carrier versus noncarrier), and were matched for the number of cases, age, and sex. The exclusion criteria for all participants were a significant neurological or psychiatric history, focal brain lesions on MRI, a history of cancer within 10 years prior to inclusion in the study, a contraindication for MRI (pacemaker, metal implants or severe claustrophobia), or exposure to ionizing radiation for research procedures above 1 mSv within the year prior to inclusion in the study.

Patients with prodromal AD were recruited from the Memory Clinic of University Hospital Leuven. Patients diagnosed with biomarker-proven AD had a cognitive deficit, were amyloid positive (CSF and/or PET scan), and maintained independence of function in daily life.

### **Image acquisition and processing**

#### **Amyloid PET**

Twenty-nine cognitively unimpaired participants underwent a dynamic ^11^C-PiB PET scan on a 16-slice Biograph Truepoint PET/CT scanner (Siemens). A low-dose CT scan of the head (11 mAs) was performed for attenuation correction, prior to PET acquisition. Administration of the ^11^C-PiB as an intravenous bolus in an antecubital vein (260.00 MBq ± 43.77 MBq [171.59 - 356.06 MBq]) was started at the same time as the start of a 60 minute dynamic PET scan. Images were reconstructed as 20 frames (4 x 15s, 4 x 60s, 2 x 150s, 10 x 300s frames) based on the OSEM algorithm (5 iterations and 8 subsets).

Thirty-four cognitively unimpaired participants underwent a static 20 minute ^18^F-NAV4694 PET scan on the same Siemens Truepoint scanner (168.79 MBq ± 15.56 MBq [136.89 - 205.2 MBq] bolus) as 4 x 300s PET frames (3 iterations and 21 subsets) starting 50 minutes post-injection.

One cognitively unimpaired participant underwent a 20 minute ^18^F-florbetaben amyloid PET scan on the same Siemens Truepoint scanner (168.79 MBq ± 15.56 MBq [136.89 - 205.2 MBq] bolus) starting 90 minutes post-injection as 4x 300s PET frames (4 iterations and 21 subsets).

All PET scans were corrected for randoms, scatter, deadtime and attenuation.

For the dynamic ^11^C-PiB scans, the first 5 minute frames were summed and this image was coregistered to the individual’s structural image. For the ^18^F-NAV4694 and ^18^F-florbetaben PET scans, the image of the first frame was used to coregister to the individual’s T1 image. The rigid body coregistration was performed using normalized mutual information in SPM12. To correct for head motion, the images (the summed first 5 minute image and all sequential frames for ^11^C-PiB and the different frames for ^18^F-NAV4694 and ^18^F-florbetaben data) were realigned using SPM12 by coregistration of each pair of consecutive frames. Frames with >6 mm translation in any direction or >6 degrees rotation in any direction were excluded for further analysis. The structural MRI was segmented in SPM12 into gray matter (GM), white matter (WM) and cerebrospinal fluid (CSF). The forward deformation field obtained during the segmentation process was used to normalize all PET frames into MNI. The cerebellar GM was used as reference region to calculate the Standard Uptake Value Ratio (SUVR) from the normalized PET images. This cerebellar reference region was derived from the Automated Anatomical Labelling atlas (labels: 91-108) and masked by the normalized subject-specific GM map with threshold for masking set at <0.3 to exclude WM contribution.^1^ For the ^11^C-PiB scans, the SUVRs were calculated between 40 and 60 minutes post-injection, while for the ^18^F-NAV4694 scans this was calculated between 50 and 70 minutes after the start of tracer injection and for the ^18^F-florbetaben scans between 90 and 110 minutes after start of the tracer injection. The mean SUVR was calculated in a global composite cortical VOI, which consisted of five bilateral cortical regions, namely frontal, parietal, anterior cingulate, posterior cingulate and lateral temporal region, defined as AAL areas: 3-10, 13-16, 23-28, 31-32, 35-36, 57-70, 81-82, 85-90.

For each tracer, Centiloid values (CL) were calculated by converting the SUVR. Similarly as De Meyer *et al.*^2^, the formula for converting ^18^F-NAV4694 was done by using an in-house procedure with an independent dataset of ^11^C-PiB and MRI images from GAAIN (Available from: <http://www.gaain.org/centiloid-project>). Calibration of the SUVR_NAV4694_ value to CL scale is a two-step process. First, linear regression between the SUVR_PiB_ with the standard CL method and SUVRs_NAV_  calculated with our own preprocessing pipeline, which resulted in a slope *m* and intercept *b*. These m and b were used to calculate the SUVR_PiB-calc_ values:

$${SUVR}_{NAV}=m*{SUVR}_{PiB}+b$$

$${SUVR}_{PiB-calc}=\frac{{SUVR}_{NAV}-b}{m}$$

Using these SUVR_PiB-calc_ values and the 0-100 anchor points obtained by assigning 0 CL as ^CU-0^SUVR_PiB_ and 100 CL as ^AD-100^SUVR_PiB_, the CL_NAV_ can be calculated:

$${CL}_{NAV}=100*\frac{{SUVR}_{PiB-calc}-{SUVR}_{PiB}^{CU-0}}{{SUVR}_{AD-100}-{SUVR}_{PiB}^{CU-0}}$$

The following formula was obtained: $CL=107.78*{SUVR}_{NAV}-114.71$


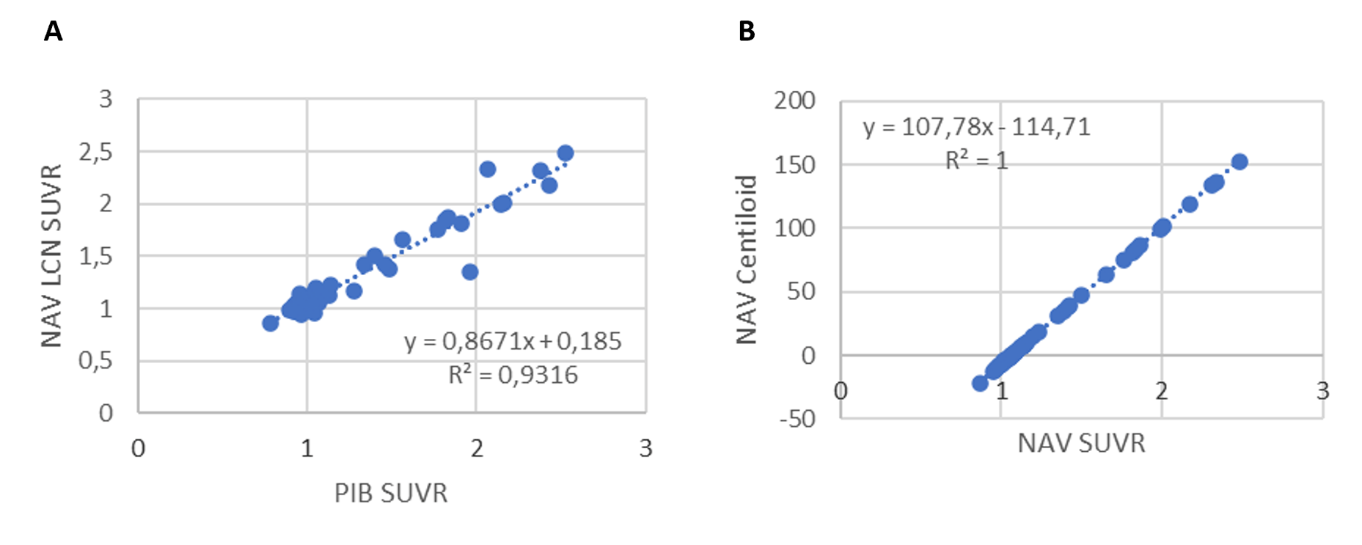


**Supplementary Fig. 1 Conversion formulas. (A)** Conversion NAV to PiB SUVR. **(B)** Conversion NAV SUVR to Centiloid.

The ^11^C-PiB and ^18^F-florbetaben SUVRs were converted to CL values using following formulas:

^11^C-PIB: $CL=132.53*SUVR(40-60)-147.64$ (method is described in Reinartz *et al.*^3^)

^18^F-florbetaben: $CL=147*SUVR\left( 90-110 \right)-166.5$ (method is described in De Meyer *et al.*^2^)

#### **Tau PET**

Images were reconstructed as 30 frames (4 x 15s, 4 x 30s, 2 x 60s, 2 x 150s, 18 x 300s) using OSEM (4 iteration and 28 subsets). Using SPM 12 running on Matlab 2021b, the first 5 minute frames of the PET data were summed and this image together with all sequential frames were realigned using SPM12 by coregistration of each pair of consecutive frames. Quality control involved control of head movement with a maximum of 6 mm translation threshold in any direction and a rotation threshold of 6 degrees in any direction. If a frame exceeded this threshold, the frame was excluded from further analysis. If too many consecutive frames exceeded this threshold, this scan was excluded from further analysis. PVC was applied using a modified Müller-Gärtner procedure.^4^ Then this image of the summed first 5 minutes was coregistered to the T1-weighted MRI (using SPM12 with rigid body transformation using normalized mutual information as criterion) and the resulting transformation was applied to all included frames. Next, the MRI was segmented into GM, WM and CSF and the forward deformation field obtained during the segmentation process was used to normalize all PET frames into MNI. The normalized PET images were entered in a Logan graphical analysis (with t* = 30min) to derive parametric distribution volume ratio (DVR) images with a subject-specific inferior cerebellar mask as reference region, defined with the individual’s T1 image and the Brainnetome atlas labels 252, 254-274 and 279-280 (cut at z = -38 as upper border).^5^ The median tau uptake values were calculated in the volumes of interest (VOI) related to neuropathologically established tau-vulnerable regions. The early metaVOI consists of the entorhinal cortex, perirhinal cortex, caudal and rostral hippocampus, lateral and medial parahippocampus and fusiform cortex and is defined by the Brainnetome atlas as labels [115-116], [109-112], [215-218], [113-114, 119,-120], [85-86, 91-92, 97-98, 105-108] and weighted for the number of voxels of each subregion. The neocortical area consists of the inferior and lateral temporal cortex and inferior and medial parietal cortex^6^ and is defined with the Brainnetome atlas labels [89:102, 103:108, 121:124, 69:80, 81:88, 135:146, 65:68, 147:155].^5^

#### **High-density EEG**

83 subjects underwent a resting-state high-density 128-electrode EEG. Data was acquired for five minutes with their eyes open, while instructed to fixate on the fixation point at the center of the screen, and five minutes with their eyes closed. All participants were tested in the morning, to account for tiredness. During the acquisition, the data quality was being controlled for drowsiness. If participants were getting tired, we took a break and restarted the five minutes acquisition. Our processing pipeline also included several data quality control steps including identification and removal of bad EEG channels and removal of eye blink artefacts.

## Supplementary results

**Supplementary table 1 Group differences of age, tau in the early metaVOI, entorhinal cortex and perirhinal cortex**

|  | **Age** | **Tau early metaVOI** | **Tau entorhinal** | **Tau perirhinal** |
| --- | --- | --- | --- | --- |
| Kruskall-Wallis test | 𝜒^2^ = 10.04, *p* < 0.001 | 𝜒^2^ = 22.44, *p* < 0.001 | 𝜒^2^ = 15.86, *p* < 0.001 | 𝜒^2^ = 23.42, *p* < 0.001 |
| **CU- VS CU+** | Age: *z* = -3.05; *p_adj_* = **0.003**  Early metaVOI: *z* = -1.25; *p_adj_* = 0.31  Entorhinal: *z* = -1.90; *p_adj_* = 0.085  Perirhinal: *z* = -2.08; *p_adj_* = 0.056 | | | |
| **CU- VS Prodromal AD** | Age: *z* = -1.85; *p_adj_* = 0.10  Early metaVOI: *z* = -4.74; ***p_adj_* < 0.001**  Entorhinal: *z* = -3.89; ***p_adj_* < 0.001**  Perirhinal: *z* = -4.78; ***p_adj_* < 0.001** | | | |
| **CU+ VS Prodromal AD** | Age: *z* = 1.05; *p_adj_* = 0.44  Early metaVOI: *z* = -2.84; ***p_adj_* < 0.001**  Entorhinal: *z* = -1.59; *p_adj_* = 0.17  Perirhinal: *z* = -2.18; *p_adj_* = 0.044 | | | |

Comparisons between three subgroups were made using Kruskall-Wallis tests (top row). Dunn’s test was used to assess the differences in each pair of groups (bottom rows). *P* -values are adjusted with Bonferroni correction for multiple comparisons. CU- and CU+ = Cognitively unimpaired amyloid negative and positive, respectively. *n* = 66.


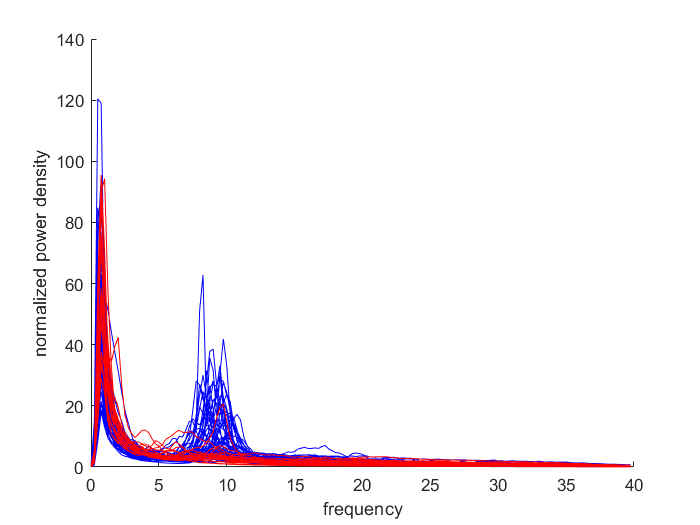


**Supplementary Fig. 2 Power analysis.** We computed the power spectrum density using the function pwelch in matlab for each electrode and using 4s epoch lengths. We then normalized for each electrode this power spectrum density by dividing by the mean power spectrum density for that electrode. Next, we averaged the normalized power spectrum density across all electrodes. In the figure this normalized power spectrum density across all electrodes is plotted for each subject. Cognitive unimpaired participants are shown in blue, patients with prodromal AD in red. *n* = 79.

**Supplementary table 2 Group differences of global clustering coefficient and characteristic path length**

|  | **Global clustering coefficient** | **Characteristic path length** |
| --- | --- | --- |
| Kruskall-Wallis test | 𝜒^2^ = 14.63, p < 0.001 | 𝜒^2^ = 16.66, *p* < 0.001 |
| **CU- VS CU+** | Clustering coefficient: *z* = -1.10; *p_adj_* = 0.41  Path length: *z* = 0.22; *p_adj_* = 1 | |
| **CU- VS Prodromal AD** | Clustering coefficient: *z* = -3.15; ***p_adj_* = 0.002**  Path length: *z* = -3.81; ***p_adj_* < 0.001** | |
| **CU+ VS Prodromal AD** | Clustering coefficient: *z* = 3.62; ***p_adj_* < 0.001**  Path length: *z* = -3.45; ***p_adj_* < 0.001** | |

Comparisons between three subgroups were made using Kruskall-Wallis tests (top row). Dunn’s test was used to assess the differences in each pair of groups (bottom rows). *P* -values are adjusted with Bonferroni correction for multiple comparisons. CU- and CU+ = Cognitively unimpaired amyloid negative and positive, respectively. *n* = 83.

**Supplementary table 3: Relationship between the global graph measures in the alpha frequency band with eyes closed and tau PET load in different regions in whole group**

|  | **Global clustering coefficient** | | | **Characteristic path length** | | |
| --- | --- | --- | --- | --- | --- | --- |
| model | β | *p_uncor_* | *p_FDR_* | β | *p_uncor_* | *p_FDR_* |
| **Tau in entorhinal** | -0.01 | 0.09 | 1.0 | 0.56 | 0.27 | 0.27 |
| age | 0.004 | 0.50 | 0.64 | 0.49 | 0.34 | 0.64 |
| sex | 0.02 | 0.13 | 0.83 | -0.89 | 0.38 | 0.60 |
| APOE4 | -0.003 | 0.83 | 0.89 | -0.45 | 0.65 | 0.89 |
| **Tau in perirhinal** | -0.02 | **0.003** | **0.016** | 1.23 | **0.013** | **0.019** |
| age | 0.007 | 0.27 | 0.64 | 0.33 | 0.51 | 0.64 |
| sex | 0.02 | 0.20 | 0.50 | -0.61 | 0.53 | 0.60 |
| APOE4 | -0.002 | 0.89 | 0.89 | -0.59 | 0.54 | 0.89 |
| **Tau in hippocampus** | -0.02 | **0.007** | **0.016** | 1.08 | **0.028** | **0.035** |
| age | 0.003 | 0.59 | 0.65 | 0.55 | 0.27 | 0.64 |
| sex | 0.02 | 0.07 | 0.50 | -1.06 | 0.28 | 0.56 |
| APOE4 | -0.003 | 0.82 | 0.89 | -0.52 | 0.59 | 0.89 |
| **Tau in parahippocampus** | -0.02 | **0.009** | **0.016** | 1.26 | **0.0098** | **0.016** |
| age | 0.005 | 0.41 | 0.64 | 0.42 | 0.69 | 0.69 |
| sex | 0.017 | 0.16 | 0.50 | -0.70 | 0.48 | 0.60 |
| APOE4 | -0.005 | 0.67 | 0.89 | -0.40 | 0.67 | 0.89 |
| **Tau in fusiform** | -0.02 | **0.006** | **0.016** | 1.38 | **0.004** | **0.016** |
| age | 0.005 | 0.40 | 0.64 | 0.42 | 0.39 | 0.64 |
| sex | 0.02 | 0.19 | 0.50 | -0.59 | 0.54 | 0.60 |
| APOE4 | -0.005 | 0.66 | 0.89 | -0.40 | 0.67 | 0.89 |

Regression model with global graph measures in the alpha frequency band in the eyes closed condition as outcome variables and tau PET load in the different regions, age, sex and APOE4 carriership as regressors. Both the regression coefficient β, uncorrected *p* value and FDR corrected *p* values are shown and were indicated in bold if significant (*p* < 0.05). *n = 66.*

**Supplementary table 4 Relationship between tau load in different regions with global graph measures in the three subgroups in alpha frequency band for eyes closed condition**

| **Cognitively unimpaired amyloid negative, *n* = 37** | | | | | | | | |
| --- | --- | --- | --- | --- | --- | --- | --- | --- |
|  | **Global clustering coefficient** | | | | **Characteristic path length** | | | |
|  | *ρ* | 95% CI | *p_uncor_* | covariates | *ρ* | 95% CI | *p_uncor_* | covariates |
| Entorhinal | -0.50 | -0.81 to 0.05 | 0.07 | *ρ* = -0.42; *p* = 0.17 95CI = -0.79 to 0.17 | 0.38 | -0.19 to 0.80 | 0.19 | *ρ* = 0.34; *p* = 0.28 95CI = -0.26 to 0.75 |
| Perirhinal | -0.69 | -0.89 to -0.25 | **0.008** | *ρ* = -0.69; ***p* = 0.01** 95CI = -0.90 to -0.23 | 0.43 | -0.13 to 0.78 | 0.12 | *ρ* = 0.47; *p* = 0.12 95CI = -0.11 to 0.81 |
| Hippo-campus | -0.30 | -0.71 to 0.28 | 0.30 | *ρ* = -0.37; *p* = 0.24 95CI = -0.76 to 0.23 | 0.11 | -0.45 to 0.60 | 0.72 | *ρ* = 0.23; *p* = 0.47 95CI = -0.37 to 0.69 |
| Parahippo-campus | -0.55 | -0.84 to -0.03 | **0.04** | *ρ* = -0.43; *p* = 0.16 95CI = -0.80 to 0.15 | 0.17 | -0.40 to 0.64 | 0.56 | *ρ* = 0.10; *p* = 0.77 95CI = -0.48 to 0.61 |
| Fusiform | -0.63 | -0.87 to -0.14 | **0.019** | *ρ* = -0.60; ***p* = 0.04** 95CI = -0.87 to -0.08 | 0.45 | -0.11 to 0.79 | 0.11 | ***ρ* = 0.59 *p* = 0.04** 95CI = 0.06 to 0.86 |
| **Cognitively unimpaired amyloid negative, n = 37** | | | | | | | | |
| Entorhinal | -0.02 | -0.34 to 0.30 | 0.90 | *ρ* = 0.0006; *p* = 0.997 95CI = -0.34 to 0.34 | -0.06 | -0.38 to 0.27 | 0.71 | *ρ* = -0.10; *p* = 0.59 95CI = -0.41 to 0.24 |
| Perirhinal | 0.02 | -0.31 to 0.34 | 0.93 | *ρ* = 0.003; *p* = 0.99 95CI = -0.34 to 0.34 | -0.10 | -0.41 to 0.24 | 0.57 | *ρ* = -0.12; *p* = 0.49 95CI = -0.43 to 0.22 |
| Hippo-campus | 0.006 | -0.32 to 0.33 | 0.97 | *ρ* = -0.04; *p* = 0.81 95CI = -0.37 to 0.30 | -0.16 | -0.46 to 0.18 | 0.36 | *ρ* = -0.13; *p* = 0.47 95CI = -0.44 to 0.21 |
| Parahippo-campus | -0.05 | -0.36 to 0.28 | 0.79 | *ρ* = -0.04; *p* = 0.82 95CI = -0.37 to 0.30 | -0.07 | -0.38 to 0.26 | 0.69 | *ρ =* -0.08; *p* = 0.65 95CI = -0.40 to 0.26 |
| Fusiform | -0.17 | -0.47 to 0.16 | 0.30 | *ρ* = -0.15; *p* = 0.41 95CI = -0.46 to 0.20 | 0.04 | -0.29 to 0.36 | 0.81 | *ρ* = 0.008; *p* = 0.96 95CI = -0.32 to 0.34 |
| **Prodromal AD, n = 15** | | | | | | | | |
| Entorhinal | 0.27 | -0.28 to 0.69 | 0.33 | *ρ* = 0.30; *p* = 0.31 95CI = -0.27 to 0.72 | -0.61 | -0.86 to -0.14 | **0.018** | ***ρ* = -0.62; *p* = 0.02 95CI= -0.87 to -0.14** |
| Perirhinal | -0.03 | -0.54 to 0.49 | 0.91 | *ρ* = -0.20; *p* = 0.53 95CI = -0.66 to 0.38 | -0.13 | -0.60 to 0.41 | 0.65 | *ρ* = -0.04; *p* = 0.90 95CI = -0.56 to 0.50 |
| Hippo-campus | -0.14 | -0.61 to 0.40 | 0.61 | *ρ* = -0.20; *p* = 0.51 95CI = -0.66 to 0.37 | -0.20 | -0.65 to 0.34 | 0.47 | *ρ* = -0.15; *p* = 0.62 95CI = -0.63 to 0.41 |
| Parahippo-campus | 0.025 | -0.49 to 0.53 | 0.93 | *ρ* = -0.17; *p* = 0.57 95CI = -0.65 to 0.39 | -0.05 | -0.55 to 0.47 | 0.86 | *ρ* = 0.05; *p* = 0.87 95CI = -0.49 to 0.57 |
| Fusiform | -0.06 | -0.55 to 0.47 | 0.84 | *ρ* = -0.26; *p* = 0.39 95CI = -0.69 to 0.31 | 0.03 | -0.49 to 0.54 | 0.91 | *ρ* = 0.14; *p* = 0.64 95CI = -0.42 to 0.63 |

Spearman ρ correlations between tau PET in the different regions and global graph measures in the cognitively unimpaired amyloid negative subgroup, cognitively unimpaired amyloid positive subgroup and prodromal AD subgroup. On top the results for the cognitively impaired group (*n* = 14), in the middle the results for the cognitively unimpaired amyloid negative group (*n* = 37) and below the results for the prodromal AD group (*n* = 15). The left column indicates each time the Spearman correlation coefficient, followed by the 95% confidence interval and the uncorrected *p* value, the right column indicates the partial correlation coefficients *ρ*, uncorrected *p* values and 95% confidence interval after adjusting for age and sex.

**Supplementary table 5 Relationship between the global graph measures in the alpha frequency band with eyes closed and amyloid Centiloid load, sex and age in the cognitively unimpaired group**

|  | **Global clustering coefficient** | | **Characteristic path length** | |
| --- | --- | --- | --- | --- |
| model | β | *p* | β | *p* |
| Amyloid (CL) | -0.0002 | 0.16 | 0.01 | 0.39 |
| age | 0.01 | 0.13 | 0.19 | 0.70 |
| sex | 0.04 | **0.002** | -2.43 | **0.01** |

Regression model with global graph measures in the alpha frequency band in the eyes closed condition as outcome variables and amyloid load in Centiloids, age and sex as regressors. Both the regression coefficient β and *p* values are shown and were indicated in bold if significant (*p* < 0.05). CL = Centiloid. *n* = 51.

**Supplementary table 6 Relationship between tau load in early metaVOI with global graph measures in the whole group in the alpha frequency band for the eyes open condition and in the theta frequency band eyes closed condition**

| **Alpha frequency eyes open** | | | | |
| --- | --- | --- | --- | --- |
|  | **Global clustering coefficient** | | **Characteristic path length** | |
| model | β | *p* | β | *p* |
| **Tau in early metaVOI** | -0.009 | 0.13 | 0.66 | 0.21 |
| Age | 0.007 | 0.21 | -0.02 | 0.98 |
| Sex | -0.002 | 0.85 | -0.43 | 0.69 |
| APOE4 | -0.0009 | 0.94 | 0.56 | 0.59 |
| **Theta frequency eyes closed** | | | | |
| **Tau in early metaVOI** | -0.006 | 0.20 | 0.23 | 0.72 |
| Age | 0.006 | 0.23 | -0.58 | 0.38 |
| Sex | -0.001 | 0.89 | -0.01 | 0.99 |
| APOE4 | -0.003 | 0.75 | -1.02 | 0.42 |

Regression model with global graph measures in the alpha frequency band in the eyes open condition (top) or theta frequency band in the eyes closed condition (bottom) as outcome variables and tau PET load in the early metaVOI, age, sex, and APOE4 carriership as regressors. Both the regression coefficient β and *p* values are shown and were indicated in bold if significant (*p* < 0.05). *n* = 66.

**Supplementary table 7 Relationship between tau load in early metaVOI with global graph measures in the whole group in the alpha frequency band for the eyes closed condition with 64, 32 and 24 electrodes**

| **64 electrodes** |  | |  | |
| --- | --- | --- | --- | --- |
|  | **Global clustering coefficient** | | **Characteristic path length** | |
| model | β | *p* | β | *p* |
| **Tau in early metaVOI** | -0.02 | **0.002** | 1.45 | **0.004** |
| Age | 0.004 | 0.50 | 0.47 | 0.35 |
| Sex | 0.02 | 0.13 | -0.71 | 0.48 |
| APOE4 | -0.002 | 0.83 | -0.45 | 0.65 |
| **32 electrodes** |  |  | |  |
| **Tau in early metaVOI** | -0.02 | **0.0005** | 1.61 | **0.004** |
| Age | 0.001 | 0.79 | 0.45 | 0.43 |
| Sex | 0.02 | 0.13 | -0.56 | 0.62 |
| APOE4 | -0.01 | 0.33 | -0.69 | 0.53 |
| **24 electrodes** | | | | |
| **Tau in early metaVOI** | -0.02 | **0.0004** | 1.50 | **0.01** |
| Age | 0.005 | 0.32 | 0.46 | 0.44 |
| Sex | 0.01 | 0.20 | -0.68 | 0.57 |
| APOE4 | -0.01 | 0.21 | -0.75 | 0.51 |

Regression model with global graph measures in the alpha frequency band in the eyes closed condition as outcome variables and tau PET load in the early metaVOI, age, sex and APOE4 carriership as regressors. Both the regression coefficient β and *p* values are shown and were indicated in bold if significant (*p* < 0.05). *n* = 66.

**Supplementary table 8 Correlations between tau load in early metaVOI and global graph measures in the subgroups in the alpha frequency band for the eyes closed condition with 64, 32 and 24 electrodes**

| **64 electrodes** | | | | | | | |
| --- | --- | --- | --- | --- | --- | --- | --- |
|  |  | **Global clustering coefficient** | | | **Characteristic path length** | | |
|  |  | Correlation coefficient | 95 % CI | *p* | Correlation coefficient | 95% CI | *p* |
| Early metaVOI | CU- | -0.16 | -0.46 to 0.17 | 0.34 | -0.05 | -0.37 to 0.28 | 0.78 |
|  | CU+ | -0.60 | -0.86 to -0.10 | **0.026** | 0.36 | -0.21 to 0.75 | 0.21 |
|  | Prodromal AD | -0.25 | -0.68 to 0.30 | 0.37 | -0.007 | -0.52 to 0.51 | 0.98 |
| **32 electrodes** | | | | | | | |
| Early metaVOI | CU- | -0.13 | -0.43 to 0.20 | 0.44 | -0.04 | -0.35 to 0.29 | 0.84 |
|  | CU+ | -0.48 | -0.81 to 0.07 | 0.08 | 0.32 | -0.26 to 0.73 | 0.27 |
|  | Prodromal AD | -0.40 | -0.76 to 0.14 | 0.14 | 0.02 | -0.50 to 0.53 | 0.95 |
| **24 electrodes** | | | | | | | |
| Early metaVOI | CU- | -0.08 | -0.39 to 0.25 | 0.65 | -0.05 | -0.37 to 0.28 | 0.77 |
|  | CU+ | -0.41 | -0.77 to 0.15 | 0.15 | 0.30 | -0.27 to 0.72 | 0.30 |
|  | Prodromal AD | -0.43 | -0.77 to 0.10 | 0.11 | -0.07 | -0.56 to 0.46 | 0.80 |

Spearman ρ correlations between tau PET in early metaVOI and global graph measures in the three subgroups for 64, 32 and 24 electrodes. *p* values are shown and were indicated in bold if significant (*p* < 0.05).

**Supplementary table 9 Factor analysis**

|  | **Factor 1** | **Factor 2** |
| --- | --- | --- |
| Eigenvalue | 2.91 | 2.14 |
| Variance explained (%) | 29.1 | 21.4 |
| Cumulative variance explained (%) | 29.1 | 50.5 |
| BSRT TR | **0.88** | 0.31 |
| BSRT DR | 0.83 | 0.20 |
| AVF | 0.37 | 0.62 |
| LVF | 0.24 | 0.40 |
| AVLT TL | 0.79 | 0.43 |
| AVLT DR | 0.61 | 0.23 |
| BNT | 0.18 | **0.68** |
| RPM | 0.38 | 0.52 |
| PALPA 49 | 0.10 | 0.63 |
| TMT B/A | -0.25 | -0.30 |

Factor analysis on whole group (n = 83) with 10 variables. Factor loadings per test in column 2 and 3. Highest factorloading per factor in bold. For abbreviations: see the abbreviation list. Empirical chi-square 15.27, *p* <0.95, Comparative Fit index = 0.99 (>0.9), RMSEA index = 0.072, Bartlett’s test *p* = 4.07e-57, KMO = 0.83.

## Supplementary References

1. Tzourio-Mazoyer N, Landeau B, Papathanassiou D, et al. Automated anatomical labeling of activations in SPM using a macroscopic anatomical parcellation of the MNI MRI single-subject brain. *Neuroimage*. 2002;15(1):273-289. doi:10.1006/nimg.2001.0978

2. De Meyer S, Schaeverbeke JM, Verberk IMW, et al. Comparison of ELISA- and SIMOA-based quantification of plasma Aβ ratios for early detection of cerebral amyloidosis. *Alzheimer’s Res Ther*. 2020;12(1):1-16. doi:10.1186/s13195-020-00728-w

3. Reinartz M, Gabel S, Schaeverbeke J, et al. Changes in the language system as beta amyloid accumulates. *Brain*. Published online 2021.

4. Müller-Gärtner HW, Links JM, Prince JL, et al. Measurement of radiotracer concentration in brain gray matter using positron emission tomography: MRI-based correction for partial volume effects. *J Cereb Blood Flow Metab*. 1992;12(4):571-583. doi:10.1038/jcbfm.1992.81

5. Fan L, Li H, Zhuo J, et al. The Human Brainnetome Atlas: A New Brain Atlas Based on Connectional Architecture. *Cereb Cortex*. 2016;26(8):3508-3526. doi:10.1093/cercor/bhw157

6. Jack CR, Andrews JS, Beach TG, et al. Revised criteria for diagnosis and staging of Alzheimer’s disease: Alzheimer’s Association Workgroup. *Alzheimer’s Dement*. 2024;20(8):5143-5169. doi:10.1002/alz.13859
